# Malignant choroidal melanoma with vitreous seeds: supported by histopathology and field emission scanning electron microscopy study

**DOI:** 10.22336/rjo.2023.31

**Published:** 2023

**Authors:** Dipankar Das, Obaidur Rehman, Kasturi Bhattacharjee, Harsha Bhattacharjee, Manab Jyoti Barman, Surjendu Maity, Dipankar Bandyopadhyay

**Affiliations:** * Department of Ocular Pathology, Uveitis and Neuro-Ophthalmology, Sri Sankaradeva Nethralaya, Guwahati, Assam, India; ** Department of Ophthalmic plastic & reconstructive surgery and facial aesthetics, Sri Sankaradeva Nethralaya, Guwahati, Assam, India; *** Department of Vitreo-Retina, Sri Sankaradeva Nethralaya, Guwahati, Assam, India; **** Centre for Nanotechnology and Department of Chemical Engineering, Indian Institute of Technology, Guwahati, Assam, India

**Keywords:** choroidal melanoma, enucleation, uveal melanoma, vitreous seeding, electron microscopy

## Abstract

**Aim:** To report an exceptionally rare case of malignant choroidal melanoma with vitreous seeding, supported by histopathological and field emission scanning electron microscopic (FESEM) studies.

**Case report:** A 58-year-old male with painless diminution of vision in his left eye for past 1 month was found to have a brown retrolental mass lesion on slit lamp examination in the left eye. Detailed fundus examination revealed choroidal melanoma in the left eye with pigmented seeds extending into the vitreous cavity and associated exudative retinal detachment. Ocular imaging was consistent with the diagnosis.

**Results:** The eyeball was enucleated and the tumor was considered as stage IIB (AJCC 8th edition classification). Metastatic workup of the patient was negative. One half of the eyeball was subjected to field emission scanning electron microscopy to further study the nature and appearance of vitreous seeds.

**Discussion:** Vitreous seeding in choroidal melanoma has been reported only in a handful of cases in literature. Histopathological confirmation of vitreous seeds was done in our case and morphological detailing was performed using FESEM study.

**Conclusions:** Treatment naïve choroidal melanoma can very rarely have vitreous seeds. Early enucleation in such cases carries a favorable prognosis.

## Introduction

Uveal melanoma is the most common intraocular tumor in adults, and choroidal melanomas represent 80% of all diagnosed cases [**1**-**10**]. Choroidal melanoma has varied presentations with characteristic findings on clinical examination and imaging [**1**-**10**]. On very rare occasions, it can rupture the Bruch’s membrane and invade through the retina, leading to vitreous seeding [**1**,**3**-**5**]. 

We report a case of an elderly patient with a mushroom-shaped melanoma, and vitreous seedings. The clinical diagnosis was supplemented with histopathology, immunohistochemistry and field emission scanning electron microscopy (FESEM) study.

## Case report

**Fig. 1:**
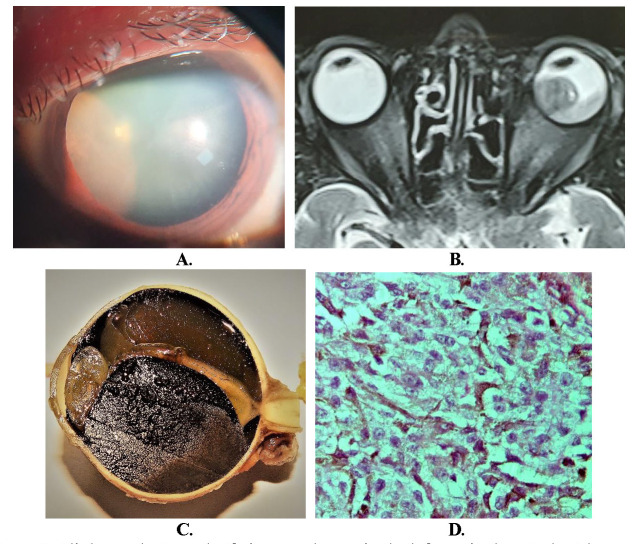
**A.** Slit lamp photograph of pigmented mass in the left eye in the retrolental area on dilatation of pupil; **B.** T2-weighted Magnetic Resonance Imaging (MRI) of brain and orbit in axial view showing a mushroom shaped hypointense large malignant choroidal melanoma; **C.** Pigmented choroidal mass involving a portion of the ciliary body associated with exudative retinal detachment. The sclera appears normal; **D.** Mixed cell melanoma on histopathology with prominence of epithelioid cells (H&E, x 40)

A 58-year-old Indian male presented with diminution of vision in the left eye (OS) for the last one month. The best corrected visual acuity was 20/ 20, N6 in the right eye (OD) and hand movement close to face in the left eye (OS). A relative afferent pupillary defect (RAPD) was noted in OS on pupillary examination. Intraocular pressures were 10 mmHg in OD and 11 mmHg in OS respectively. On slit-lamp examination, the anterior chamber was quiet, but a brown colored mass lesion in the retrolental area was seen in OS (**Fig. 1a**). Dilated fundus examination of OS revealed a large choroidal melanoma with surrounding exudative retinal detachment (ERD) and scattered seeds in the vitreous cavity. Anterior segment, as well as posterior segment examinations, were unremarkable in OD. B-scan ultrasonography of OS showed a moderately reflective mushroom-shaped mass arising from the choroid, with choroidal excavation and protruding anteriorly into the vitreous cavity. A note of acoustic hollowing and shadowing was made. An associated moderately high reflective membrane echo with a corrugated pattern, suggestive of ERD, was also observed. The optic nerve was normal in appearance. Magnetic resonance imaging (MRI) of the brain and orbit revealed a large intraocular mass arising from the posterior medial aspect of the left globe, seen as hyperintense in T1-weighted scan and hypointense in T2-weighted scan (**Fig. 1b**). An anterior extension of the mass into the vitreous cavity was also noted. No obvious local extension could be observed. Complete blood counts, liver function tests, computerized tomography of the thorax and ultrasound of the whole abdomen were within normal limits. Due to large tumor size and unwillingness of the patient for brachytherapy, a decision to enucleate the eyeball with placement of ball implant was considered appropriate. After a detailed informed consent, the surgery was performed under general anesthesia.

## Results

**Fig. 2:**
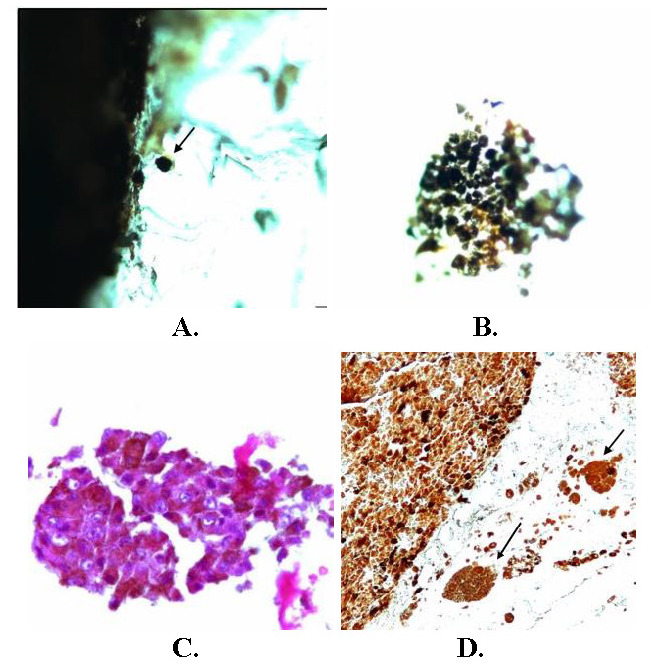
**A.** Pigmented vitreous seeds in grossing (marked with black arrow); **B.** Enlarged view of isolated pigmented seed in the vitreous cavity; **C.** Isolated vitreous seed in hematoxylin-eosin stained slide (x 40); **D.** Immunohistochemistry for HMB-45 showing vitreous seeds (marked with arrow; ScyTek Laboratories, Logan, UTAH, USA; x 20)

The enucleated eyeball (OS) was grossly normal in appearance and measurements, but a transillumination defect was observed. The eyeball was sectioned vertically and a deep anterior chamber was noted. The iris and crystalline lens were normal. A large pigmented tumor, measuring 14.39 mm in height and 18.92 mm in basal diameter, was observed in the vitreous cavity (**Fig. 1c**). The pigmented tumor (in bleached preparation) was observed arising from the choroid and had an associated ERD. On histopathological examination of the tumor, mixed cells consisting of spindle A, B and epithelioid cells with fascicular pattern were observed (**Fig. 1d**). A portion of the ciliary body was also involved by the tumor. Pigmented macrophages were noted in the tumor. On histopathology, cut end of the optic nerve was normal. A diagnosis of large malignant choroidal melanoma with mixed cellular patterns and choroidal was made. No extrascleral spread of the tumor was noted. Grossly, perilesional choroidal melanoma seeding was observed in the vitreous, which was confirmed with hematoxylin-eosin stain and by immunohistochemistry positivity for Human melanoma black-45 (HMB-45) stain (**Fig. 2**).

**Fig. 3:**
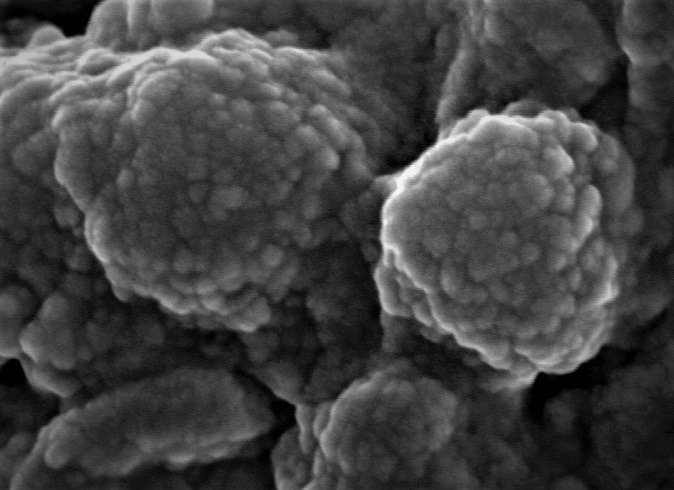
Field emission scanning electron microscopy photograph of vitreous seeds (x 10000)

One half of the sample was prepared for FESEM analysis at the Indian Institute of Technology, Guwahati. The tissue containing choroidal melanoma seeds was placed over a glass slide, which was further placed on the sample holder, using carbon tape. The set-up was subsequently dehydrated overnight at room temperature inside a vacuum desiccator, following which it was covered with platinum using a plasma sputter. The FESEM (JEOL, JSM-7610F, Japan) was then used to study the surface morphology of the choroidal melanoma seeds. Under electron microscopy, the seeds were seen as spherical bodies with cell surface irregularities (**Fig. 3**).

The postoperative period was uneventful. A detailed metastatic work up showed negative results. The tumor was graded by American Joint Committee on Cancer (AJCC) 8th edition classification as pT2b. A histological grading of G2: Mixed cells, spindle A and B with few epithelioid cells was made, with AJCC prognostic group - stage IIB.

## Discussion

Choroidal melanoma is a malignant intraocular growth observed in the adult population [**1**-**10**]. The size of the lesion can be variable and, as the choroidal melanoma enlarges, it can lead to rupture of the Bruch’s membrane and invasion into the retina, leading to extrusion of pigmented seeds into the vitreous cavity [**1**,**3**-**5**]. There are only few reported cases of such seedings in the scientific literature [**1**,**3**-**5**]. Like retinoblastoma seeds, which have been studied in detail until present, choroidal melanoma seeds also require an extensive study in the future [**1**,**3**-**5**]. Location and size of the tumor are important [**1**,**3**-**5**]. A large tumor or involvement of ciliary body (hidden under the iris and delayed diagnosis) carry a poorer prognosis. Our case also involved a part of the ciliary body. Melanophagic pigments may be observed in the vitreous in advanced melanomas, which are necrotic in nature, unlike the ones observed in our case (viable seeds) [**4**]. An important differential diagnosis of vitreous seeding by uveal melanoma is the presence of pigment-laden macrophages in the vitreous cavity. Many cases of pigment in the vitreous cavity may represent pigment-laden macrophages and not true seeding by the uveal melanoma. Histopathology can differentiate between the two entities in a definitive manner, by showing actual tumor seeds as clustered melanoma cells in a unit. Brachytherapy, a modality of treatment in uveal melanoma, can also cause shedding of pigmented seeds into the vitreous cavity [**4**,**5**]. Our patient was a treatment naïve case and had received no such treatment before the enucleation surgery. One study suggested that the early enucleation of the eye with choroidal melanoma having vitreous seeding may be beneficial with respect to the overall prognosis [**5**]. Our patient did not have any extra-scleral involvement and mixed type of cells were observed histopathologically. FESEM study was utilized in our case to show irregularly studded melanocytic cells over the seedings, which were of variable sizes and clustered around the main pigmented mass. No retinal pigment cells were observed over the seedings. The authors have also tried imaging vitreous seeds by utilizing Optical Coherence Tomography (OCT) in other cases and, in our experience, OCT can identify seeds more than 80 microns in size.

## Conclusion

Vitreous seeding is an uncommon phenomenon in choroidal melanoma. To the best of our knowledge, the present report is the tenth case of vitreous seeds in choroidal melanoma and the first case that has incorporated the FESEM study. 

**Conflict of Interest statement**

The authors state no conflict of interest.

**Informed Consent and Human and Animal Rights statement**

Consent to publish the case report was not obtained. This report does not contain any personal information that could lead to the identification of the patient.

**Authorization for the use of human subjects**

Ethical approval: The research related to human use complies with all the relevant national regulations, institutional policies, is in accordance with the tenets of the Helsinki Declaration, and has been approved by the review board of the Department of Ophthalmic plastic & reconstructive surgery and facial aesthetics, Sri Sankaradeva Nethralaya, Guwahati, Assam, India.

**Acknowledgements**

1. Sri Kanchi Sankara Health and Educational Foundation.

2. Prof. Panna Deka and Mr. Apurba Deka, Ocular Pathology Department, Sri Sankaradeva Nethralaya, Guwahati, Assam, India.

**Sources of Funding**

The authors state no funding or grant support.

**Disclosures**

None.

**Commercial relationships**

None.

**Competing interests**

None.

**Authorship**

All authors attest that they meet the current ICMJE criteria for authorship.
